# The future of combination immunotherapy in oesophageal adenocarcinoma

**DOI:** 10.3389/fimmu.2023.1217132

**Published:** 2023-07-14

**Authors:** Maria Davern, Noel E. Donlon

**Affiliations:** ^1^ Department of Medical Oncology, Dana-Farber Cancer Institute, Harvard Medical School, Boston, MA, United States; ^2^ Department of Surgery, Trinity St. James’s Cancer Institute, Trinity Translational Medicine Institute, St. James’s Hospital, Trinity College Dublin, Dublin, Ireland; ^3^ Department of Upper GI Surgery, Beaumont Hospital, Dublin, Ireland

**Keywords:** oesophageal, immunotherapy, PD-1, CTLA-4, TIGIT, neoadjuvant, chemotherapy, chemoradiotherapy

## Introduction

Oesophageal adenocarcinoma (OAC) is now the predominant subtype of oesophageal cancer in Western countries and its incidence is rapidly increasing due to rising levels of obesity (1). OAC principally affects the distal oesophagus and gastroesophageal junction (2). The main risk factors for OAC include obesity, gastro-oesophageal reflux disease and the pre-malignant condition Barrett’s Oesophagus (3). OAC has one of the poorest long-term outcomes of all solid tumors with an overall 5 year survival rate in the region of 20% (4). Despite advances in endotherapy and surgical approaches, a significant proportion of patients present at an advanced and inoperable stage due to the indolent nature of the disease (5). In the contemporary era in which combination therapies complementing surgery are standard in the curative approach to patients presenting with locally advanced disease, this has improved survival in OAC patients (6, 7). However, significant pathological response rates to the standard of care first-line chemotherapy (FLOT: 5-fluorouracil, leucovorin, oxaliplatin, docetaxel) and chemoradiotherapy (CROSS: carboplatin, paclitaxel and 1.8 Gy radiation over 23 fractions) regimens are approximately 20% (1). Recent FDA approvals of immune checkpoint blockers (ICBs) targeting the PD-1 axis has sparked new possibilities for designing more tolerable and effective treatment options for OAC patients (8, 9). The effectiveness of PD-1/PD-L1 blockade as monotherapies or in combination with FLOT has been limited to a subset of patients, a phenomenon typically observed across several cancer types (8, 9). A multitude of mechanisms conferring both primary and acquired resistance have been documented in other tumor types which have been useful in forming different hypotheses behind the lack of efficacy for PD-1/L1 blockade in OAC (10, 11). To broaden the benefits of PD-1/L1 blockade to a wider spectrum of OAC patients, a biology-first approach must be adopted to successfully identify the right immune checkpoint to target in combination with PD-1/L1 blockade. It is widely accepted that the primary function of these evolutionary conserved immune checkpoint proteins is to regulate immune homeostasis by fine-tuning the immune response to ensure sufficient immune activation to eradicate an invading or foreign entity but also to prevent overstimulation of the immune system avoiding collateral damage to normal tissues that might lead to the development of autoimmune diseases (12). Immune checkpoints also inhibit activation of autoreactive T cells that could promote autoimmunity (12). These well-characterized functions of immune checkpoint proteins have formed the central dogma for many years (13). Following T cell activation, inhibitory immune checkpoint receptors are upregulated on the surface of T cells (14). Due to chronic antigen stimulation and the inhospitable hypoxic and nutrient deprived tumor microenvironment several inhibitory immune checkpoint receptors such as PD-1, TIM-3, LAG-3 and CTLA-4 are upregulated on T cells that cooperatively inhibit T cell proliferation, cytokine production and function creating an exhausted T cell phenotype (15, 16). Blockade of inhibitory immune checkpoints reinvigorates anti-tumor T cells, unleashing powerful anti-tumor immune responses, increasing T cell proliferation and production of anti-tumor cytokines to mediate eradication of the tumor (17). Immune checkpoint receptors are also expressed by other immune cells such as natural killer cells (18), macrophages (19), dendritic cells (20–22), myeloid-derived suppressor cells (23). Ultimately, intrinsic immune checkpoint signaling inhibits their anti-tumor function, propagating pro-tumorigenic properties, inducing apoptosis in natural killer cells or polarizing these cell types toward a more tumor-promoting phenotype such as anti-inflammatory macrophages (24).

More recently, novel immune-independent functions have also been discovered for immune checkpoint proteins in OAC (25) and other cancer types (25). It is well-known that tumor cells upregulate inhibitory immune checkpoint ligands such as PD-L1 to facilitate immune escape (26). However, we now know that tumor cells such as OAC cells can also express inhibitory immune checkpoint receptors and *via* tumor cell-intrinsic signaling these inhibitory immune checkpoint receptors can promote various hallmarks of cancer in OAC cells such as a cancer stem-like phenotype, DNA damage repair (27), proliferation (27, 28), chemo(radio)therapy resistance (27, 29, 30) and altered metabolism (25). These immune checkpoints include the well-known PD-1 and PD-L1 as well as novel immune checkpoints such as TIM-3, TIGIT, LAG-3 and A2aR (25, 27–29). These immune-independent functions of immune checkpoints have also been uncovered in many other cancer types outside of OAC including melanoma (31), colorectal, cervical (31, 32), lung (33) and head and neck cancer (34, 35).

## Recent successes for PD-1 blockade in OAC

PD-1 blockade has undoubtedly been the most successful immunotherapy in the clinic and is anticipated to form an integral pillar for cancer care in the future. However, identifying what anti-cancer therapies will work best with PD-1 blockade and extend its durable efficacy to more patients is a significant clinical challenge. A multitude of resistance mechanisms to PD-1 blockade have been documented, with significant inter-patient variability (10, 11, 36). Clinicians and patients are in need of a functional precision biomarker or test that can 1) predict response and 2) assign the ‘right’ therapy to the patient that will successfully convert a PD-1-blockade ‘non-responder’ into a ‘responder’. Currently, this remains a significant clinical challenge in the field.

PD-1 blockade has demonstrated efficacy in OAC and has led to two FDA approvals, one in the neoadjuvant setting and another in the adjuvant setting. The CheckMate 649 trial led to the FDA approval of nivolumab in combination with the FLOT chemotherapy regimen after demonstrating an improved overall survival of 13.8 vs. 11.6 months in advanced gastric cancer, gastroesophageal cancer and OAC (9). Pre-clinical findings highlighted that FLOT directly upregulated PD-L1 on the surface of stem-like OE33 and SK-GT-4 cell lines *in vitro* and on OAC-donor-derived T cells *ex vivo*, possibly creating a therapeutic vulnerability that was capitalized on by using anti-PD-1 therapy. PD-1 blockade inhibited both immune-independent and dependent mechanisms in OAC cells. Firstly, PD-1 blockade directly enhanced FLOT-mediated killing of OE33 and SK-GT-4 cells and decreased the percentage of stem-like ALDH^+^ OAC cells *in vitro* (27, 28). In addition, anti-PD-1 enhanced OAC-donor lymphocyte killing of OE33 cells. These findings highlight the complementary effects of FLOT and PD-1 inhibition, which collectively propagate anti-tumor immunity and the cytotoxicity of first-line chemotherapy providing possible mechanistic insights behind the beneficial results observed in the CheckMate 649 trial (28, 37).

Furthermore, the landmark CheckMate 577 phase III trial led to the approval of nivolumab in resected oesophageal or gastroesophageal junction cancer patients (8). Nivolumab significantly improved disease free survival compared to placebo. (22.4 vs. 11.0 months) (8). Research in several cancer types (38–41) including oesophageal cancer (42) revealed that the Th2-like wound healing response triggered by the surgical wound promotes a systemic pro-metastatic and immunosuppressive response. The function of nivolumab is to enhance an anti-tumor Th1-like response (43). Understandably, use of PD-1 blockade in the post-operative setting may help subvert the surgery-induced pro-tumorigenic effect and propagate a stronger anti-tumor immune response enhancing immune surveillance and prolonging disease recurrence. A recent study (44) showed that the systemic effects of surgery decreased lymphocyte cytotoxicity and production of anti-tumor cytokines in PBMCs derived from OAC patients. The ability of OAC-derived lymphocytes to kill OE33 OAC cells *in vitro* was significantly decreased in PBMCs obtained 1 and 7 day post-surgery compared PBMCs obtained pre-operatively (on the day of surgery - day 0) or 6 weeks post-surgery. The use of PD-1 blockade treatment enhanced lymphocyte-mediated killing of OE33 cells and increased production of anti-tumor cytokines in lymphocytes, overcoming the surgery-mediated suppression of lymphocyte cytotoxicity. This phenomenon was similarly observed in renal cell carcinoma patients who received adjuvant pembrolizumab or placebo and the progression-free survival at 24 months was 77.3% vs. 68.1% (45).

## Next generation immune checkpoint blockers are on the horizon

Considering the multitude of immune checkpoint proteins that exist, this suggests that some immune checkpoints may likely have overlapping functions and/or have tissue and organ specific functions. Therefore, examining the immune checkpoint expression profile along the normal-pre-malignant-adenocarcinoma disease sequence would be a logical first step to uncover rational immune checkpoint targets in the context of OAC. The immune checkpoint expression profile of normal oesophageal epithelial cells (HET1A), pre-malignant Barrett’s oesophagus (BO) cells (QH cells) and OAC cells (OE33 and OE19 cells) were examined (28). Protein expression analysis revealed that PD-1 and TIGIT were expressed on 30-40% of normal oesophageal epithelial cells, that decreased to 20-30% on pre-malignant BO cells and were further downregulated to 5-10% in OAC cells *in vitro* (28). It is very surprising that non-cancerous non-immune cells also express immune checkpoint receptors. This phenomenon has also been observed in gastric (46) and cervical tissues (32), whereby normal non-cancerous gastric and cervical cells express TIM-3 immune checkpoint receptor. Considering the homeostatic immunoregulatory role of immune checkpoint proteins, this may indicate that PD-1 and TIGIT could play an important role in maintaining immune homeostasis in oesophageal tissue. The binding of TIGIT to its ligands expressed on pro-inflammatory macrophages (47) or dendritic cells (48) promotes a regulatory phenotype and PD-1 can bind with PD-L1/L2 expressed on Th1 cells or myeloid cells to enhance an anti-inflammatory phenotype (49). Strategies like this would be useful in controlling the inflammatory response induced by gastric reflux in the oesophagus, a risk factor for the development of OAC (50). If TIGIT and PD-1 are prominent players in oesophageal tissue for controlling immune activation, then targeting both TIGIT and PD-1 in tandem to remove the brakes on anti-cancer immunity may be an effective combination to test in OAC.

Combination ICB is gaining further interest for treating OAC patients. The approval of dual anti-PD-1 plus anti-CTLA-4 blockade for front-line treatment of unresectable/metastatic oesophageal squamous cell carcinoma patients has ignited hope that the effectiveness of combination ICB may also translate to OAC patients (51). Higher levels of circulating CD45^+^CTLA-4^+^ cells correlated with advanced stage disease and a poor response to neoadjuvant chemo(radio)therapy regimens in OAC patients (15). Notably, first-line FLOT chemotherapy regimen upregulated CTLA-4 on the surface of T cells which might be a potential mechanism for promoting disease progression and suppressing anti-tumor immunity leading to inferior responses to neoadjuvant regiments (37). Use of anti-CTLA-4 in combination with FLOT to exploit this therapeutic vulnerability and prevent immune dysfunction could be an effective strategy to boost treatment success and warrants further investigation. *Ex vivo* findings demonstrated that combining anti-CTLA-4 with nivolumab increased OAC donor lymphocyte production of IFN-γ more substantially than either agent alone (16). CheckMate 032 phase III clinical trial is currently ongoing testing the efficacy of combination nivolumab-ipilimumab in OAC patients (51). Early data suggest that the dual combination is superior to single agent nivolumab and improved 12-month progression-free survival (17 vs. 8%) (51).

Careful evaluation of the immunosuppressive effects of the tumor microenvironment on the efficacy of immune checkpoint blockade will be important in maximizing the therapeutic benefit. Tumor microenvironmental studies revealed that tumor-associated acidosis abrogated the ability of dual nivolumab-ipilimumab to enhance IFN-γ production in OAC donor PBMCs *ex vivo* (16). Notably, this acidic environment upregulated multiple immune checkpoints including PD-1, PD-L1, CTLA-4, TIM-3 and LAG-3 on the surface of T cells (16). The authors observed that high LAG-3 expression on T cells was significantly associated with a decreased T cell production of anti-tumor TNF-α (16). It is plausible to postulate that perhaps the upregulation of LAG-3 and Tim-3 on T cells under acidic conditions may be a mechanism to hinder the efficacy of dual nivolumab-ipilimumab treatment. An increased expression of LAG-3 on the surface of tumor-infiltrating CD8^+^ T cells also correlated with advanced stage disease in OAC patients (37). Further investigation is required to elucidate whether blockade of LAG-3 and/or TIM-3 in combination with dual nivolumab-ipilimumab may overcome immune checkpoint blockade failure in acidic tumors. Optimistic findings for the success of anti-LAG-3 as a complementary partner to PD-1 blockade has been reported in melanoma patients (52). The phase II/III RELATIVITY-047 trial led to the momentous FDA approval of relatlimab (anti-LAG-3) in combination with nivolumab in untreated advanced melanoma patients (52). The median progression-free survival was 10.1 months vs. 4.6 months (52). With a comparable efficacy profile to dual nivolumab-ipilimumab therapy and a substantially improved toxicity profile (59% vs. 21%), these results offer optimism in the quest to find tolerable and more effective ICB combinations to treat OAC patients in the future (52). Trials testing dual LAG-3 plus PD-1 blockade are warranted in OAC patients.

Upregulation of TIM-3 has been identified as a mechanism of acquired resistance to PD-1 blockade in several solid tumors and so, may also likely be a relevant and druggable mechanism of resistance in OAC (53). The AMBER phase I trial testing the safety and efficacy of cobolimab (anti-TIM-3) plus nivolumab/dostarlimab (anti-PD-1) in a range of solid tumors (melanoma, mesothelioma and neuroendocrine carcinoma) confirmed its safety profile and has moved to phase II. Early data reported that patients in the monotherapy arms experienced no clinical benefit (54). However, patients in the combination arms achieved partial responses. These are promising findings in support of the potential efficacy of dual anti-PD-1 plus anti-TIM-3 combinations, clinical testing will need to be performed in OAC to determine if this combination could benefit OAC patients.

FLOT chemotherapy regimen reportedly upregulated A2aR on the surface of OE33 and SK-GT-4 cell lines (28). Administering A2aR blockade directly induced OAC cell death as a monotherapy and elicited an additive effect in combination with FLOT chemotherapy (27). Clinical trials have yet to be carried out in OAC patients to test the effectiveness of A2aR blockade as a monotherapy or in combination with other ICBs. Encouraging trial data reported in refractory renal cell carcinoma patients that combined A2aR antagonism with anti-PD-L1 therapy induced partial responses in 11% of patients (55). Clinical trials will be necessary to determine if this paired combination could improve efficacy of anti-PD-1 therapy in OAC.

OE33 and OE19 cells were cultured under glucose deprivation and hypoxic conditions (0.5% O_2_) to mimic the inhospitable tumor microenvironment (25). Under such conditions TIGIT was upregulated on the surface of OAC cells, which the authors postulated was an adaptive survival strategy to withstand harsh conditions (25). To test this, OE33 and OE19 cells were treated with anti-TIGIT under these hostile conditions and it was observed that blockade of TIGIT induced OAC cell death suggesting that TIGIT may provide OAC cells with some form of survival advantage (25). These pre-clinical findings laid down convincing evidence for TIGIT as a novel immunotherapeutic target in OAC. Data from a phase Ib trial testing anti-TIGIT (tiragoliuab) plus atezolizumab (anti-PD-L1) in heavily pre-treated metastatic oesophageal cancer patients achieved an objective response rate of 27.8% with an acceptable toxicity profile (7 patients - oesophageal squamous cell carcinoma, 3 patients - oesophageal adenocarcinoma, 1 - patient neuroendocrine carcinoma) (56).

Like TIGIT, PD-1 expression on OE33 and OE19 cells reportedly increased under glucose deprived hypoxic conditions (25). Considering that PD-1 blockade was shown to induce cell death in OE33 and SK-GT-4 cell lines in normal culture conditions as well as enhancing OAC cell death in combination with the FLOT regimen (27), it was hypothesized that PD-1 blockade may play a survival role in OAC cells under glucose deprived hypoxic conditions. Surprisingly PD-1 blockade enhanced OAC cell survival under these conditions (25). In the same study under normal culture conditions, PD-1 blockade enhanced basal respiration and glycolytic reserve in OAC cells. This finding may suggest that PD-1 blockade may promote a more metabolically ‘fitter’ phenotype that might allow OAC cells to better survive in a nutrient deprived and hypoxic environment. However, that remains speculative, and the precise mechanisms remain to be elucidated (25). The findings from this study support a rationale to combine anti-TIGIT with anti-PD-1 blockade in OAC. Both are known to enhance anti-tumor T cell immunity, and in the case of a nutrient deprived hypoxic tumor TIGIT blockade could counteract the pro-survival benefit provided by anti-PD-1 under those precise conditions. **Table 1** summarizes the immune-dependent and -independent functions of immune checkpoints and their clinical status in OAC.

**Table 1:** The immune-dependent and -independent functions of immune checkpoint pathways in OAC and the pre-clinical or clinical status of pharmacological agents designed to target these pathways in OAC.

Immune checkpoint Pathway	Immune-dependent functions in OAC	Immune-independent functions in OAC	Pre-clinical/clinical status in OAC
**PD-1/PD-L1/PD-L2**	Promotes Th1 cell dysfunction and an anti-inflammatory or regulatory T cell phenotype (57).	Promotes a cancer stem-like phenotype, DNA damage repair, proliferation, decreases chemotherapy sensitivity and radiosensitivity in OAC.	FDA approved adjuvant nivolumab in OAC (8). FDA approved neoadjuvant nivolumab + FLOT in OAC (9).
**TIGIT/PVR**	Promotes Th1 cell dysfunction, regulatory T cell, macrophage and dendritic cell phenotype (58).	Alterations in tumor metabolism, promotes tumor cell survival in OAC.	Pre-clinical testing of anti-TIGIT *in vitro* in OAC and clinical testing in OAC (25, 56).
**TIM-3/Galectin-9**	Promotes Th1 cell dysfunction and induces apoptosis.	Unknown	Only clinical testing of anti-TIM-3 in other cancer types and not in OAC yet.
**LAG-3/MHC II**	Promotes Th1 cell dysfunction and exhaustion (59).	Unknown	Only clinical testing of anti-LAG-3 in other cancer types and not in OAC yet.
**A2aR-Adenosine**	Promotes Th1 cell dysfunction and exhaustion (60).	Promotes tumor cell viability and reduces chemotherapy sensitivity in OAC.	Pre-clinical testing of anti-A2aR *in vitro* in OAC (27).
**CTLA-4-CD80/86**	Inhibits T cell priming (61).	unknown	Clinical testing of anti-CTLA-4 in OAC (61).

PD-1, programmed death-1; PD-L2, programmed death ligand-1; PD-L2, programmed death ligand-2; TIGIT, T cell immunoglobulin and ITIM domain; PVR, Poliovirus receptor; TIM-3, T cell immunoglobulin and mucin domain 3; LAG-3, Lymphocyte activation gene 3; MCH II, major histocompatibility complex II; A2aR, adenosine A2a receptor, CTLA-4, cytotoxic T lymphocyte antigen-4.

## Safety profile of ICB and standards of care in OAC

**Figure 1:**
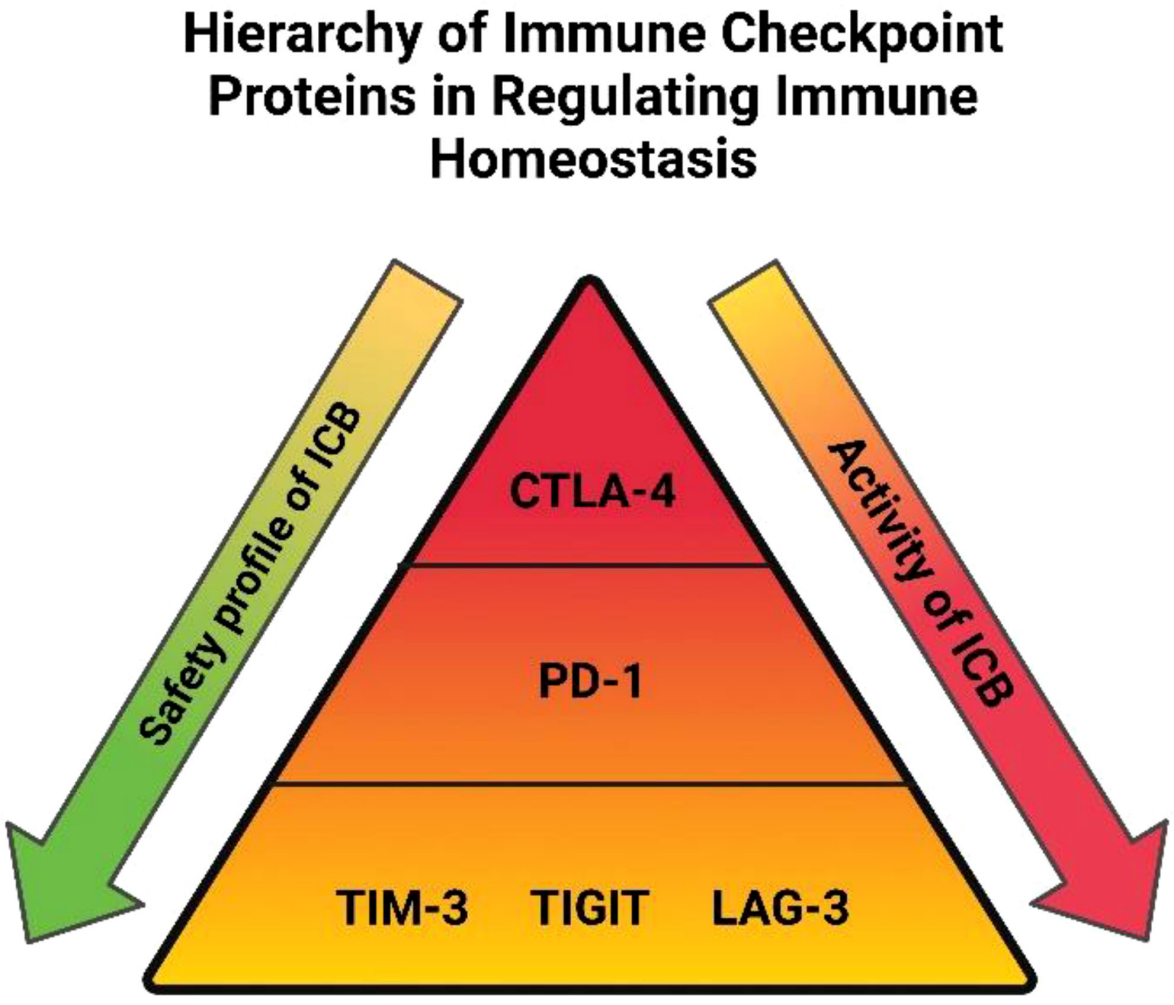
Hierarchy of immune checkpoint proteins in regulating immune homeostasis: Immune checkpoints are placed in a hierarchical order based on their importance relating to immune homeostasis, safety profile and pharmacological activity with regards to blocking each individual immune checkpoint protein. A higher tier in the pyramid denotes immune checkpoints with a more extensive role in maintaining immune tolerance in the body. Blockade of immune checkpoints residing in the lower tiers is accompanied with a more favorable safety profile than those in higher tiers. The pharmacological activity associated with blocking immune checkpoint proteins is also depicted, blockade of CTLA-4 has shown efficacy in only a subset of patients compared with PD-1 blockade, whose efficacy extends to a much larger proportion of patients. The efficacy of TIM-3, TIGIT and LAG-3 blockade isn’t as well investigated but preliminary findings suggest their combination with PD-1 blockade will be just as effective as combination with CTLA-4 blockade but with a greater safety profile.

Emerging evidence in cancer types outside of OAC suggest that the incidence of immune-related adverse events following treatment with ICB might be associated with clinical outcomes, this has yet to be elucidated in OAC (62). However, the benefit from ICB is tempered by the emergence of toxic side effects which involve diverse organs, has varying biology, onset time, and severity (63). When designing ICB combinations for OAC patients it will be important to appreciate the accompanying toxicity profiles with blockade of multiple immune checkpoints. The degree of toxicities associated from blockade of the most common immune checkpoint proteins are depicted in a hierarchical pyramid in **Figure 1** (64). CTLA-4 is at the top of the pyramid, regulating early T cell proliferation primarily in the lymph nodes and its blockade is associated with often severe immune related adverse events (65). PD-1 regulates T cell proliferation later in the immune response mainly in peripheral tissues and is in the middle of the pyramid with more tolerable adverse events compared with CTLA-4 blockade (65). TIGIT, TIM-3 and LAG-3 are more specialized, possessing unique functions that they exert at specific tissue sites regulating distinct aspects of immunity (64). These three immune checkpoints are located at the bottom of the pyramid and their blockade is associated with a much greater safety profile than PD-1 and CTLA-4 (64). Indeed, a deeper insight into the precise function and expression profile of immune checkpoints in OAC will be critical in guiding the rational selection of the ‘right’ immune checkpoint protein to target with PD-1/L1 blockers to harness the power of the anti-cancer immune system, analogous to the concept of precision medicine. Early clinical trials suggest that these more specialized immune checkpoints namely LAG-3 in combination with PD-1 blockade exhibit a superior safety profile compared with PD-1 plus CTLA-4 blockade with equivocal anti-tumor activity (**Figure 1**) (52). The advent of the next generation of ICBs and their early success in clinical trials in other cancer entities creates optimism that finding the ‘right’ ICB to combine with PD-1/L1 blockade to improve therapeutic efficacy in OAC might be a fast-approaching possibility (52). Another prudent observation when considering the future of current standards of care for OAC patients, is that ICB has a more tolerable safety profile than conventional chemo(radio)therapy regimens. Nivolumab has been shown to possess a much greater safety profile in OAC with only 10% of patients receiving neoadjuvant nivolumab experiencing grade 3 or 4 immune-related adverse events in the ATTRACTION-2 trial (66) and only 10% of patients receiving adjuvant nivolumab discontinued treatment in the CheckMate 577 trial (8). This is in stark contrast to only 35% of OAC patients receiving all 8 cycles of the first-line FLOT chemotherapy regimen due to grade 3 and 4 treatment-related toxicities (67). This means that OAC patients could experience a better quality of life while receiving immunotherapy over the current cytotoxic regimens that comprise the standard of care. The safety data for the use of ICB in combination with current standards of care is limited. In the CheckMate 649 trial which led to the approval of nivolumab with FLOT chemotherapy, an increase in grade 3 and 4 treatment-related adverse events compared with FLOT alone were observed (59% vs. 44%) (68). According to a met-analysis of 4,379 patients with a variety of solid tumours combining ICB (PD-1/L1 or CTLA-4 blockade) with chemotherapy, there was an increased incidence of grade 3 and 4 treatment-related adverse events and consequently discontinuation of treatment observed (69). However, mortality rates were not increased suggesting that combination chemoimmunotherapy approaches and careful management of treatment-related toxicities is instrumental to prevent treatment-related mortality (69).

## Conclusion

Several trials testing the effectiveness of anti-PD-1 as a dual checkpoint approach with novel ICBs such as anti-TIGIT, anti-LAG-3 or anti-TIM-3 are ongoing. The results are eagerly awaited with anticipation that these novel ICB combinations will represent the next generation of immunotherapies to benefit OAC patients.

## Author contributions

MD and NED conceptualized and wrote the manuscript. All authors contributed to the article and approved the submitted version.
